# Unifying Subicular Function: A Predictive Map Approach

**DOI:** 10.1101/2024.11.06.622306

**Published:** 2024-11-07

**Authors:** Lauren Bennett, William de Cothi, Laurenz Muessig, Fábio R Rodrigues, Francesca Cacucci, Tom J Wills, Yanjun Sun, Lisa M Giocomo, Colin Lever, Steven Poulter, Caswell Barry

**Affiliations:** 1 Department of Cell and Developmental Biology, University College London, London, UK; 2 Department of Neuroscience, Physiology & Pharmacology, University College London, London, UK; 3 Institute of Behavioural Neuroscience, University College London, London, UK; 4 Department of Neurobiology, Stanford University School of Medicine, Stanford, CA, USA; 5 Howard Hughes Medical Institute, Stanford University School of Medicine; Stanford, CA, USA; 6 Department of Psychology, Durham University, Durham, UK.

## Abstract

The successor representation has emerged as a powerful model for understanding mammalian navigation and memory; explaining the spatial coding properties of hippocampal place cells and entorhinal grid cells. However, the diverse spatial responses of subicular neurons, the primary output of the hippocampus, have eluded a unified account. Here, we demonstrate that incorporating rodent behavioural biases into the successor representation successfully reproduces the heterogeneous activity patterns of subicular neurons. This framework accounts for the emergence of boundary and corner cells; neuronal types absent in upstream hippocampal regions. We provide evidence that subicular firing patterns are more accurately described by the successor representation than a purely spatial or boundary vector cell model of subiculum. Our work offers a unifying theory of subicular function that positions the subiculum, more than other hippocampal regions, as a predictive map of the environment.

## Introduction

The hippocampal formation is intimately linked to episodic memory and spatial cognition^1-3^ - functions believed to rely on distinct populations of spatially modulated neurons. Most notably, these include CA3/1 place cells^4^ and medial entorhinal cortex grid cells^5^, as well as several other cell-types distributed through the hippocampus and associated regions^6-9^. Collectively, these neurons, which constitute a representation of self-location, are held to form a cognitive map^10^ that supports flexible navigation and reasoning in both physical and abstract spaces^11,12^.

Compared to CA3/1 and entorhinal cortex, subiculum — the primary output structure of the hippocampus — has received comparatively little attention. This oversight is surprising given its computational potential: subiculum contains a larger number of principal neurons with more diverse morphology than CA3 and CA1 combined^13^, coupled with extensive recurrent connectivity^14,15^. Moreover, subiculum holds a privileged position in the connectome, with dense projections to an extensive cortical and subcortical network that includes anterior thalamic nuclei, retrosplenial, medial prefrontal, and entorhinal cortices^13,16,17^.

To a certain extent, this relative neglect can be attributed to the absence of a clear and unifying computational role for subiculum. Subicular principal neurons exhibit a diverse menagerie of spatial responses^6^, with authors often choosing to emphasise one distinct aspect of their observed activity. Amongst these, boundary vector cells are perhaps the best known. Initially theorised as an allocentric input to place cells^18^, neurons matching this description were subsequently identified in subiculum^19,20^. However, these cells lie downstream of primary CA3/1 projections^17^, raising the possibility that they may be derived from place cell activity, rather than contributing to it. Boundary vector cells are characterised by elongated firing fields that run parallel to boundaries and generally align with the surrounding environmental geometry^20,21^. A subsection of these fields also generate ‘trace’ responses that persist after barrier removal^8^. These characteristics have been construed as demonstrating a potential role for subiculum in representing and recalling environmental geometry and physical affordances^8,19,20,22^.

Further studies have highlighted the role of subiculum in representing movements and trajectory sequences. For example, subicular neurons have been reported to represent the current axis of travel^23^, as well as composite signals of place and head orientation^24^, specific trajectories^25^, running speed^25,26^, and head direction^23,26^. Adding to this complexity, Sun *et al.*^9^ recently identified subicular neurons that represent corners — both concave and convex — within an environment.

Unlike subiculum, the simple hippocampal place code has attracted considerable attention from theoreticians proposing normative and mechanistic models^27-30^. Recently, predictive coding has emerged as a powerful framework for understanding hippocampal function and the generation of neural responses in associated regions^31-36^. In particular, the successor representation, which calculates expected future state occupancies^37^, accounts for how place fields are shaped by both environmental boundaries and animal behaviour^32,38^. Importantly, the successor model consolidates evidence that agent behaviour greatly influences spatial representations^39-41^, and explains navigational and search biases observed in humans and rodents performing spatial tasks^42,43^.

While the successor representation can model spatial responses as long-run predictions over discrete locations^32^, it can also be learnt over amalgams of sensory information termed ‘features’ that correspond to a more plausible neural representation of state^38,44^. Building on this, recent work has shown that biological computations within the hippocampus are sufficient to approximate successor learning^45-47^. Thus, spike-timing-dependent plasticity^48^ (STDP) over sequences of place cell firing, ordered by theta sequences, can sweep into subiculum^28,49,50^ and provide a physiological mechanism that rapidly encodes transitions between fields.

Although in previous work the successor representation has been deployed as a model of CA3/1, we here propose a reconceptualisation based on the position of the hippocampus atop a multi-modal sensory hierarchy. Thus, place cell responses can be viewed as generalised amalgams of information from diverse modalities^51-53^ – corresponding to neural representations of spatial or non-spatial states depending on the available sensory input. We posit that STDP acting on theta sweeps over these states, or equivalent computations, yields successor features in the downstream subiculum that align with its unique anatomical and physiological properties. Furthermore, because rodents exhibit stereotyped behaviours, such as thigmotaxic running and corner-dwelling, we hypothesise that successor features based on biologically realistic trajectories, using place cell activations as basis features, will more closely match the statistics, features, and appearance of subicular representations than those found in other hippocampal regions.

Here, we test this proposal by comparing predictions from the successor representation model to two electrophysiological and one calcium imaging dataset recorded from rodent subiculum^8,9,21^ and hippocampus^54,55^. We find that successor features, derived from place cell basis features and trained on real rodent trajectories, closely resemble subicular spatial responses. Specifically, this simple model generates corner responses^9^ and border activations that correspond to boundary vector cells^19,20^. We demonstrate that the boundary responses emerging from the successor representation framework better match the population statistics of subicular boundary cells than predictions derived from the boundary vector cell model^18^. Furthermore, we use representational similarity analysis (RSA)^54^ to quantify that a successor representation model based on rodent trajectories fits a composite dataset comprised of three subicular experiments better than alternative models. Collectively, these findings support the first unified, computational account of subicular function; positioning it as a predictive map of the environment, derived from hippocampal inputs.

## Results

**Fig. 1:**
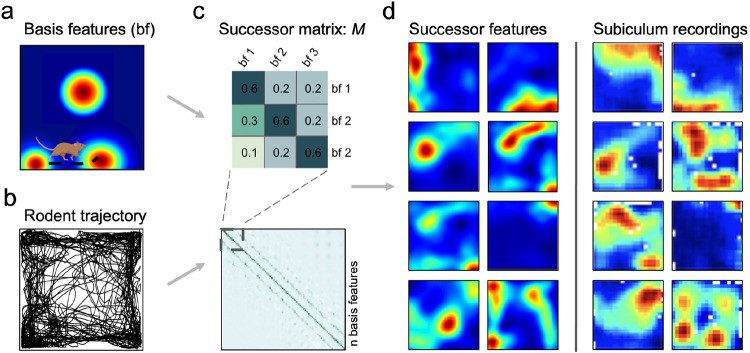
Model pipeline. (**a**) Biological basis features (place fields) were sampled along real rodent trajectories (**b**) (N=2458) in square environments in order to learn the successor matrix, M (**c**). A representative single trial trajectory in a 100cm square (**b**) highlights rodent thigmotaxis and preference for corner dwelling; with 85.7% occupancy in the perimeter versus the equally-sized inner area (mean distance of animal to nearest wall=11.8cm). Once trained, successor features are formed by the matrix multiplication of M with the population vector of basis features. (**d**) Exemplar successor features generated from rodent trajectories (left columns) closely resemble rodent subicular responses (right columns), here recorded by Sun *et al.* 2024.

To investigate whether subiculum implements a successor representation over hippocampal states, we modelled CA1 as a set of spatially-modulated ‘basis features’, $\phi (s_{t})$, that resemble the observed statistics of hippocampal place cells^53^. Thus, in a rectangular environment, these sparse basis features vary with location, $s_{t}$, and are instantiated as thresholded 2D Gaussian fields (see Methods) that increase in size towards the centre of the environment and compress orthogonal to boundaries (Fig.1a; key results replicated with symmetric, uniform sized Gaussian fields in Supplementary Figure 6). The longest distance rodent trajectory from each of the three datasets^8,9,21^ was down-sampled to 10-12 Hz (Fig.1b; Methods) and the successor matrix, M, (Fig.1c) was updated at each time point according to the temporal-difference learning rule:

(1) $$M\leftarrow M+\alpha [\phi (s_{t})+\gamma \psi (s_{t+1})-\psi (s_{t})]{\phi (s_{t})}^{T}$$

where:

(2) $$\psi (s_{t})=M\phi (s_{t})$$

are the corresponding successor features.

The population activity of successor features at a given time, $\psi (s_{t})$, constitutes a predictive code that collectively captures long-run expectations about upcoming spatial states. Thus, these expectations recapitulate rodent behavioural biases, such as a proclivity to run along boundaries (thigmotaxis) and dwell near corners. The resultant successor features extend along walls and into corners (Fig.1d left); ultimately resembling the firing patterns of subicular neurons (Fig.1d right).

### Boundary responses

**Fig. 2:**
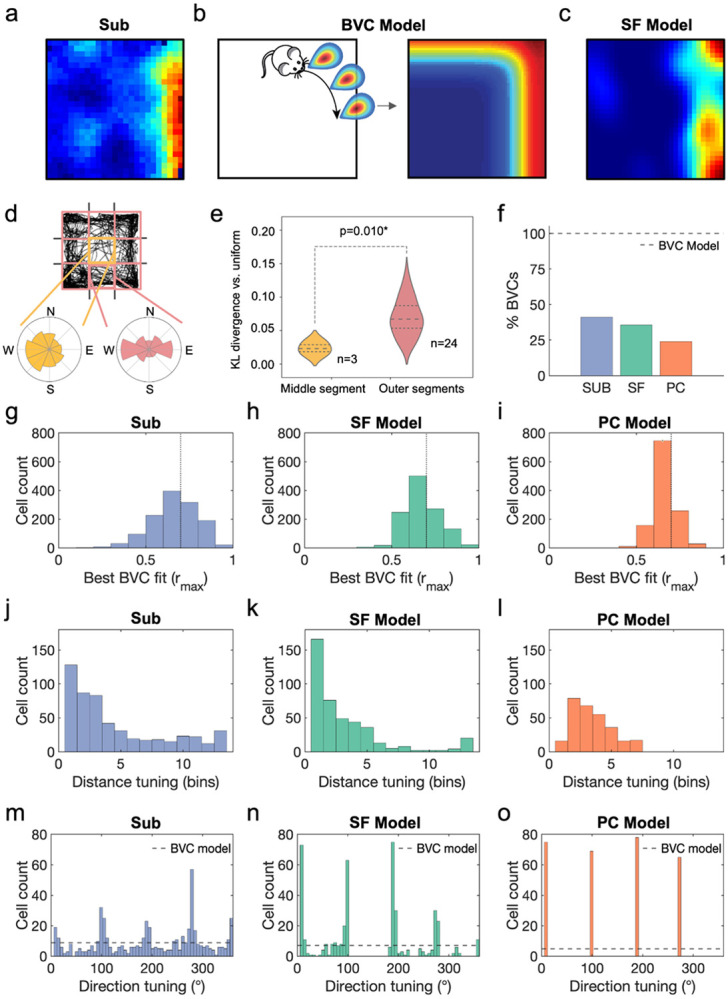
Successor features produce subicular boundary representations. (**a**) Boundary-responsive cells in rodent subiculum have previously been explained by (**b**) the boundary vector cell model as having receptive fields tuned to environmental boundaries at certain directions and distances. Conversely, the successor feature model (**c**) predicts that behavioural biases near walls produce similar boundary responses in the resulting successor features. This is due to (**d**) trajectory heading directions being more heterogeneous in the central portion of the environment than at the perimeter adjacent to walls, where trajectories are constrained both directionally and by anxiety behaviours such as thigmotaxis, measured by (**e**) the Kullback-Leibler divergence of trajectory headings vs uniform distribution (pairwise t-test middle vs outer segments, using the longest distance trajectory from each dataset, t(2)=−9.97, p=0.010). (**f**) Fitting the boundary vector cell (BVC) model to subicular data (SUB), successor features (SF) and a Gaussian place cell control model (PC) identified 40.1%, 35.6% and 23.9% of cells as having boundary vector tuning, respectively. Model fits were similar between (**g**) subiculum data and (**h**) the successor feature model (t-test following Fisher z-transform: t(2483)=0.057,p=0.955), with both producing significantly better fits than (**i**) the place cell control model (SUB vs PC, t(2483)=5.67, p<0.001; SF vs PC, t(2483)=6.54, p<0.001). (**j**) Subicular boundary cells were found to over-represent boundary responses proximal to environmental walls, better matching (**k**) the successor feature model than (**l**) the place cell control (pairwise t-test on absolute probability density residuals between SUB-SF vs SUB-PC, t(12)=3.672, p=0.003). Crucially, (**m**) vectorial responses in subiculum were clustered around directions orthogonal to the walls in a square arena (quadrupled, wrapped directional tunings to test four-fold symmetrical clustering: Rayleigh test for non-uniformity, v=90.9, p<0.001), as predicted by (**n**) the successor feature model (quadrupled, wrapped direction tunings, Rayleigh test for non-uniformity, v=229, p<0.001; Watson-Williams test of circular means, SUB vs SF, F_1,953_=0.54, p=0.462), while the canonical boundary vector cell model predicts evenly-distributed tunings^18,19^ (dashed line) and (**o**) the place cell control model only detects vectorial tunings perpendicular and slightly distal to boundaries.

We first set out to investigate whether successor features provide a better account of subicular boundary responses than prior theories - specifically the boundary vector cell model^18-20^. Boundary-responsive neurons in subiculum (Fig.2a) are explained in the boundary vector cell model as having receptive fields that are tuned at certain distances and directions to environmental boundaries^18^ (Fig.2b). Conversely, the successor feature model (Fig.2c) proposes that boundary responses arise from predictable patterns in animal movement; particularly thigmotaxic running alongside walls^56^. To quantify these behavioural biases, we segmented each environment into a 3x3 grid (Fig.2d top) and calculated the polar histogram of heading directions in each segment (Fig.2d bottom). Trajectory heading directions in segments adjacent to boundaries were significantly less heterogeneous than those in the central portion of the environment (Fig.2e; pairwise t-test between Kullback-Leibler divergence from uniform distribution for the longest distance trajectory from each trajectory used to generate successor features, t(2)=−9.97, p=0.010). This means that basis features spanning the same stretch of wall become highly predictive of one another, giving rise to successor features that diffuse along boundary edges.

The boundary vector cell and successor feature models make competing and testable predictions about the morphology of boundary responses in subiculum. Specifically, canonical versions of the boundary vector cell model propose that vector responses evenly span all directional tunings and encompass distance tunings sufficient to cover the local environment^18,19^, in order to provide a comprehensive and boundary-centred representation of allocentric space. Conversely, rodents’ proclivity to run adjacent and parallel to environmental boundaries means that the successor feature model predicts subicular responses that align proximal to environmental walls.

To test these two predictions, we adopted the procedure of Muessig *et al.*^21^ and fit the boundary vector cell model to subicular^8,9,21^ and successor feature data. We simulated N=3120 boundary vector cells in a square environment for each of the three independent datasets^8,9,21^, and used vectorial direction tunings that spanned 0-354° in 6° increments and preferred distance tunings that covered 4-52% of the environment’s dimension in 4% increments. Using an exhaustive search that maximised the Pearson r correlation between a candidate rate map and the best fitting boundary vector cell model rate map, we classified and compared the boundary vector cell (BVC) model fits to subicular data, the successor feature model (SF) built on the longest distance rodent trajectory from each dataset, and the Gaussian place cell control model (PC). Following^21^, a population threshold of r=0.7 was used, classifying 40.1% of N=1285 cells in subiculum as fitting the boundary vector cell model (Fig.2f), compared to 35.6% and 23.9% of cells in the successor feature and place cell models, respectively (N=1200 for both). Best fitting correlation values were similar between subicular data (Fig.2g) and the successor feature model (Fig.2h; t-test following Fisher z-transform: t(2483)=0.057, p=0.955) with both producing significantly better fits than the place cell control model (Fig.2i; SUB vs PC, t(2483)=5.67, p<0.001; SF vs PC, t(2483)=6.54, p<0.001).

In line with our previous work^21^, subicular boundary responses over-represented short-distance tunings (Fig.2j), and provided a better match to the successor feature model (Fig.2k) than the place cell control (Fig.2l; pairwise t-test on magnitude of probability density residuals between SUB-SF vs SUB-PC, t(12)=3.67, p=0.003). Further, following ^21^, subicular vectorial responses were clustered near directions orthogonal to environmental walls, such as the cardinal axes of a square arena (Fig.2m). We quantified this four-fold symmetrical clustering using the Rayleigh test on the quadrupled, wrapped directional tunings from the boundary vector cell model fits (Rayleigh test for non-uniformity, v=90.9, p<0.001). These characteristics were matched by the successor feature model (Fig.2n; Rayleigh test for non-uniformity, v=405, p<0.001; Watson-Williams test of circular means, SUB vs SF directional tunings, F_1,953_=0.541, p=0.462), where stereotyped behaviour along walls, coupled with more heterogeneous behaviour away from boundaries (Fig.2d,e), generated an absence of long-range and off-axis boundary responses. Conversely, the place cell control model was only matched to perpendicular vectorial tunings (Fig.2o; Rayleigh test for non-uniformity, v=287.0, p<0.001) and less proximal (Fig.2l) boundary distances.

Notably, the successor feature model predicts that individual biases in animal trajectories will propagate into inhomogeneities in spatial fields. Indeed, such models have previously been used to explain the backwards skewing of CA1 place fields against the direction of travel on linear tracks^32,47,57^. In the successor feature model, this anticipatory skew occurs because earlier locations are predictive of an animal’s future position along the track. If subicular representations do arise from a successor framework learnt over CA1 basis features, they should also exhibit path-dependent skewing - a core property of successor features^32,47^. Specifically, we would expect subicular fields to exhibit greater path-dependent skewing between consecutive trials than CA1 place cells recorded in the same environment. We reasoned that this effect should be particularly evident in border-responsive neurons, given that rodents have stronger behavioural biases near environmental boundaries.

**Fig. 3:**
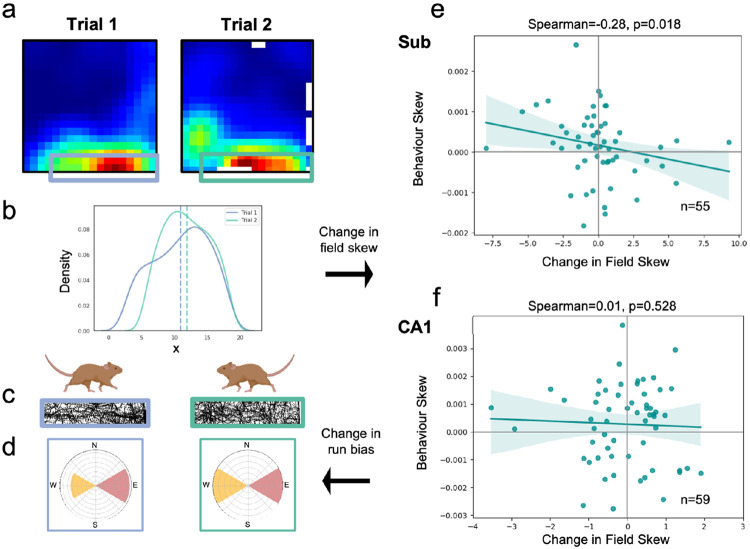
Boundary-tuned subicular responses are skewed by behavioural biases. The successor representation framework predicts that spatial firing fields will skew against an agent’s dominant axis of travel. To quantify this, (**a**) boundary-responsive CA1 and subicular fields were isolated and (**b**) averaged onto a single dimension, parallel to the adjacent wall. (**c,d**) The change in the directional bias of rodent runs through these fields was then regressed against the change in their centre of mass between consecutive trials. (**e**) Consistent with the predictive successor framework, subicular fields skewed significantly against rodents’ dominant axis of travel. (**f**) Notably, this backwards skew was not evident, under these conditions, in corresponding CA1 fields.

To test this proposition, we identified subicular cells with firing fields that were adjacent to boundaries in two consecutive trials (Fig.3a; peak firing <=5 bins from a wall, corresponding to <7.5cm for data from ^21^ (N=42 cells) and <8.3cm from ^9^ (N=13 cells) – data from ^8^ did not include consecutive trials without other manipulations). We averaged each cell’s main firing field onto its long axis (x or y) and calculated the change in its 1-dimensional centre of mass between trials as its skew (Fig.3b). Next, we quantified the bias of rodent runs through these fields, parallel to the cardinal walls (Fig.3c,d; see Methods). For those cells that were recorded over two consecutive trials (N=55 cells, 110 ratemaps), we found that subicular fields skewed significantly against the dominant axis of travel (Fig.3e; Spearman’s r(53)=−0.28, p=0.018). The skew of CA1 fields (N=59 cells, 118 ratemaps) recorded in the same environment was not significant (Fig.3f; Spearman’s r(57)=0.01, p=0.528), likely because without specific goal-location manipulations^58,59^, behaviour in a 2-dimensional environment is less biassed and directionally constrained than on an linearised track^57^.

### Corner cells

**Fig. 4:**
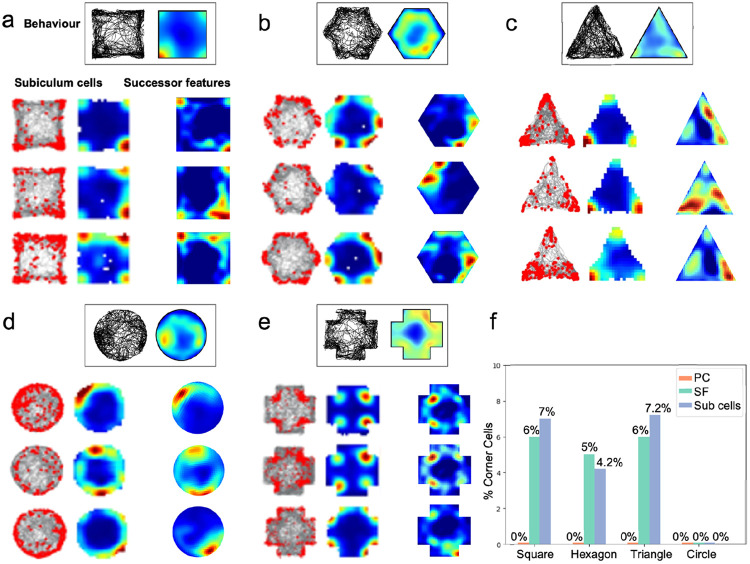
Successor features trained on real rodent trajectories exhibit subicular-like corner responses. Sun and colleagues recently characterised corner representations in subiculum as a distinct neural sub-class. (**a-e**) Top: rodent trajectories and positional heatmaps in square, hexagon, triangle, circle and cross environments, respectively. Bottom-left: subicular recordings (data from ^9^). Bottom-right: successor features generated using the same trajectories. Position heatmaps and behavioural paths highlight rodents’ preferences for running along walls and dwelling in corners. (**f**) The percentages of corner cells, defined using the ‘corner score’ metric^9^, were comparable between populations of successor features and subicular recordings, across multiple environmental geometries. Negligible place field basis features were classified as corner cells. The proportion of corner cells in the cross environment was not reported in Sun *et al.*^9^.

Neurons that fire when an animal is present at an internal corner of the environment^9^ have recently been characterised as a unique subicular subclass, distinct from boundary-responsive cells (Fig.4). We hypothesised that these corner representations could also be accounted for by a successor representation model that recapitulates animals’ behavioural biases. Specifically, when rodents dwell in adjacent corners, linked by rapid runs along the intervening walls (Fig.4a-e), the resultant successor features integrate positional information over multiple corner locations.

In order to quantify corner representations in the successor representation model, we adopted Sun and colleague’s ‘corner score’ metric^9^. Across a range of different environmental geometries and using symmetric Gaussian basis features, the successor representation model generated a strikingly similar proportion of corner cells to those recorded empirically in subiculum. For example, in a 100cm square environment, 6% of successor features were identified as corner cells compared to 7% of subicular cells (across geometries SF vs experimental: χ^2^(1, N=100)=0.20, p=0.905, Fig.4f; see Methods) - in contrast 0% of place field bases were classified as being corner responsive.

### Population comparison

**Fig. 5:**
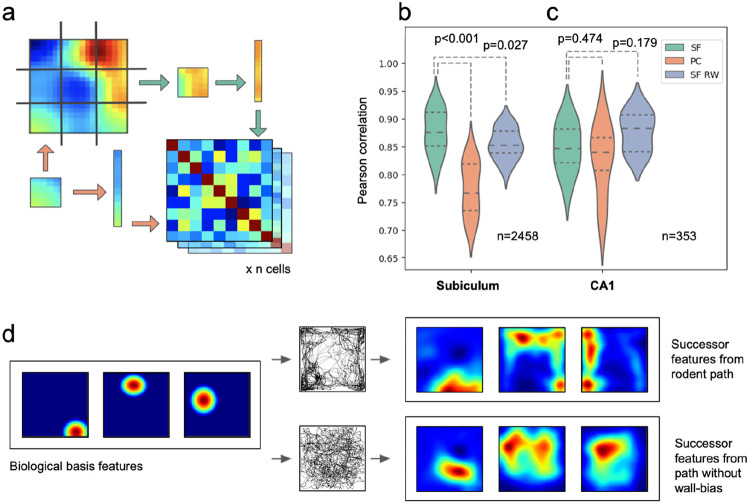
Subicular spatial responses are best described by a successor representation model trained using rodent trajectories. (**a**) Ratemaps from subicular recordings, successor features and place cell populations were each partitioned into nine sections, flattened, and cross-correlated. 9x9 correlation matrices were then averaged at either the rodent or rodent-trial level. (**b**) RSA correlations between populations of subicular cells to i) successor features based on rodent trajectories (SFs), ii) biological basis features modelled on place cells (PCs), and iii) successor features based on a biological random walk trajectory (SF RW). (**c**) Equivalent RSA results for a population of CA1 cells also recorded in the ^21^ environment are not better fit by SFs than SF RWs. Subicular responses, but not CA1 place fields, are fit better by successor features trained on real rodent trajectories. (**d**) Cartoon illustration of how wall-biassed behaviour, compared to a random walk, skews biologically inspired place cell basis features into elongated successor features, akin to subicular activations.

Finally, we used representational similarity analysis (RSA)^60^ to test whether the successor feature model better fits all subicular representations than existing computational models, agnostic of specific cell types such as boundary or corner cells. Specifically, we segmented subicular ratemaps from all three datasets into 3x3 grids, correlated within-cell ratemap activities between pairs of grid segments, and averaged across all cells in each experiment to yield a single 9x9 RSA matrix per model (Fig.5a). Our analysis used subiculum data^8,9,21^ from 9 rats and 5 mice (16 to 366 cells per animal) and CA1 data recorded from 8 rats^54,55^ (5 to 112 cells per animal) in square environments (box sides 30-100cm). We compared these condensed measures of biological population activity to similar matrices constructed for: i) Successor features (SF) based directly on real rodent trajectories (using the longest distance trial from any rat for each of the three datasets^8,9,21^); ii) Successor features trained on synthetic random walk paths^61^ with inertia but no tendency for wall-bias (thigmotaxis) (SF RW) and; iii) Biological basis features (place fields) used to derive i and ii (PC).

Ultimately, we found that the successor representation model provided a significantly better fit to subicular cells than competing models (Fig.5b; SF vs PC: 0.88 vs 0.78, t(13)=8.08, CI=(0.26,0.45), p<0.001; SF vs SF RW: 0.88 vs 0.85, t(13)=2.48, CI=(0.02,0.27), p=0.027): results that highlight the importance of animals’ behavioural biases in shaping successor features. Conversely, the successor representation framework did not yield a significantly better fit to CA1 cellular recordings than place cell basis features, or successor features generated from a random walk trajectory (Fig.5c; SF vs PC: 0.85 vs 0.83, t(7)=0.76, CI=(−0.14,0.26), p=0.474; SF vs SF RW: 0.85 vs 0.88, t(7)=−1.49, CI=(−0.29,0.06), p=0.179). This suggests that subicular cells, more so than CA1, are influenced by specific biases in animals’ trajectories, which propagate into the successor features and differentiate them from uniform random walks (Fig.5d; see Supplementary Figure 6 for consistent results using symmetric, uniform Gaussian basis features).

## Discussion

Here we present the first single, unifying model of diverse subicular responses, within the framework of predictive representations. We demonstrate that a successor framework trained on biologically plausible CA1 basis features (modelled as place fields) generates spatial responses that closely resemble those reported in rodent subiculum. Specifically, we illustrate that a subset of successor features reproduce boundary vector cell responses^19,20^ and provide a better account of biological boundary-responsive neurons than their namesake model^18^. These cells exhibit behaviour-dependent skewing, as predicted by the successor framework, thus suggesting that the boundary vector cell model might better describe entorhinal border responses^7^ than subicular activity. Our framework also accounts for subicular corner cells^9^, which we show emerge from animals’ idiosyncratic interactions with environmental geometry. At a population level, we demonstrate that the successor framework provides a compelling account of diverse subicular activity across two distinct electrophysiological and one calcium imaging dataset. Our representational similarity analyses reveal that this framework outperforms alternative models in capturing the full spectrum of subicular responses; encompassing both well-characterised cell types and more complex, mixed representations.

Thus put simply, the successor representation framework suggests that much of the complexity observed in subicular responses can be reduced to a single predictive objective^31,32^. This can be learnt over states defined by hippocampal place cells via biologically plausible processes, such as spike-timing-dependent plasticity acting over theta sequences^45-47^.

Certain aspects of known subicular data, however, are not explicitly accounted for by this framework. For instance, the duplication of boundary responses against multiple walls within or between environments^19,20^ is not directly explained, nor is the presence of distinct corner representations. Within-environment duplication, observed for drops as well as walls^8,62^, would plausibly occur if basis features duplicate between analogous locations, as observed in place fields after barrier insertion^19^ or in environments with multiple similar corridors or spaces^39,63^. The cross-environment duplication is more challenging to explain, as some proportion of place cells typically remap under these conditions^20^. We speculate that subicular networks, with their extensive recurrent connectivity^14,15^, might support compositional successor representation features that can be redeployed across environments. Such a mechanism would account for responses that generalise across contexts, such as trajectory dependent firing^23^, and the preservation of boundary vector cell characteristics across environments^8,20^. Moreover, such a mechanism offers a computational basis for the rapid and flexible adaptation of learned behaviours to novel settings—a key aspect of spatial cognition.

An implication of our work is to propose distinct roles for the two main hippocampal outputs. CA1 appears to transmit a condensed representation of states that are assembled from wide-reaching multimodal sensory inputs, while subiculum represents commonly utilised trajectories afforded by environmental geometry. This differentiation does not negate prior work proposing that CA1 place fields can be understood within a predictive framework^32,47^. Rather, it suggests a shift in the relative emphasis and temporal scope of predictive coding between these structures. The hierarchical relationship between the two regions implies that subiculum operates with a longer effective time horizon than CA1. Subiculum’s extended predictive scope may enable more complex and far-reaching spatial predictions, while CA1 encodes a more immediate representation of current and near-future states.

In conclusion, our work provides the first unifying and computational account of subicular function, positioning it as a predictive map of the environment, derived from hippocampal inputs. This framework not only explains a wide range of observed subicular responses, but also suggests new experimental directions for probing the predictive nature of subicular representations and their role in spatial cognition.

## Methods

### Neural data

We used three experimental datasets in our analysis. Dataset 1 was collected by Muessig and colleagues^21,54,55^ and contains electrophysiological recordings from male (3-6mo) lister hooded rats, freely exploring a plain 62.5x62.5cm square environment for 15 minute trials. Each rat explored the same squared environment for two to three trials per day. The subicular recordings are from three rats^21^ and the CA1 recordings are from a different eight^54,55^. Each rat was implanted with an eight-prong tetrode attached to a micro-drive. All Dataset 1 ratemaps were pre-binned into matrices for each trial. We also obtained the trajectory of each rat for each corresponding trial and environment.

In order to filter out interneurons and axons from CA1 recordings, we applied the triple filter used in Muessig *et al.*^21^, where we selected only ratemaps whose neurons had a mean firing rate less than 5Hz, whose spike width was greater than 0.3ms (peak to trough) and whose mean autocorrelation was less than 25ms. To remove the remaining non-spatial neurons, we only selected cells with a spatial information (SI) score greater than 1. We cleaned the CA1 ratemaps by removing any outer rows or columns with more than five NaNs, a procedure that we repeated twice for each ratemap. We only selected those ratemaps whose resulting shape was 25x25. We then normalised these ratemaps to sum to one and subtracted the minimum value from each entry. We then padded these ratemaps to 27x27 matrices, which resulted in 353 CA1 ratemaps covering two consecutive trials.

Next, we similarly filtered the subicular cells of Dataset 1 to include only those with mean firing rates less than 5 Hz and with a spike width greater than 0.3ms. We removed any outer rows or columns with more than five NaNs and repeated this procedure twice for each ratemap. We then normalised each ratemap to sum to one and subtracted the minimum value from each entry. We padded these ratemaps to 27x27 matrices, which resulted in 335 subicular ratemaps.

Dataset 2 was collected by Sun *et al.*^9^ and includes miniscope calcium imaging data from a total of five male and female mice freely exploring a plain 30x30cm square environment for 20 minute trials. Each mouse explored the same square environment for two trials in the same day, with at least a two hour gap between sessions. We cleaned these ratemaps by removing any outer rows or columns with more than three NaNs, a procedure that we repeated twice for each ratemap. We only selected those ratemaps whose resulting shape was 18x18. We normalised each ratemap to sum to one and subtracted the minimum value from each entry. This resulted in 1937 ratemaps which covered two consecutive trials.

Dataset 3 was collected by Poulter and colleagues^8^ and contains electrophysiological recordings from 6 male Lister hooded rats, aged 3–5 months old at implant, freely exploring a box of size 100x100cm for 20 minute trials. We filtered the pre-binned ratemaps by including only those with a mean firing rate less than 5Hz, as spike width information was not available. We cleaned these ratemaps by removing any outer rows or columns with more than three NaNs, a procedure that we repeated twice for each ratemap. We only selected those ratemaps who had the expected resulting shape of 51x51 matrices. We then normalised each ratemap to sum to one and subtracted the minimum value from each entry. This resulted in 186 ratemaps collected over one trial only.

### Successor features and biologically modelled PC

We generated three separate populations of successor features and biologically modelled basis features using RatinABox^61^ for each of the three neural datasets. First, we created an environment of the same shape as each filtered subiculum dataset (for reference, a 100x100 environment scaled to 1x1 environment). We then selected the longest distance trial trajectory from each dataset’s available rodents and down-sampled it to 10-12 Hz. We used these longest distance trajectories to generate 400 unthresholded and biologically plausible Gaussian basis features for each of the three environments. These basis features followed biologically stable statistics of place cell widths and shapes, with fields being smaller and more elongated near walls, as per Tanni *et al.*^53^:

(3) $$width=G\;(1∕H\text{-}H∕(H^{2}+wall\_distance^{2}))+W$$

where we set the minimum field width (W) to 0.053, wall height (H) to 1 and the gain level (G) to 0.74 for all successor feature populations. However, we also reproduce key results using uniform and symmetric Gaussian basis features in Supplementary Figure 6. We then normalised each basis feature’s ratemap to sum to 1, subtracted its 40th percentile, and set all negative values to 0.

To calculate the successor features, we thresholded all place cell basis features for ease of training and then used these to update a 100x100 transition matrix, M, as per Equation (1). We only updated the matrix, M, whenever the agent’s velocity was >0.0001 between timesteps (corresponding to 0.025cm per timestep). We used a learning rate of 0.002 and a gamma of 0.995 for all three populations to optimise convergence. We generated all successor features using a smoothing sigma of 1.8. We normalised all successor features and thresholded them at the 40th percentile, setting any negative values to 0.

We generated random-walk successor features for each of the three environments using the same 400 basis features, and a trajectory that began randomly within each environment and followed a biologically plausible movement sequence, without thigmotaxis, as defined in RatinABox^61^. We ensured that the average displacement for each random walk was comparable to the average displacement of the rodent for each of the three datasets.

### Boundary vector cell model fits

To fit the boundary vector cell model, we adopted the approach of Muessig *et al.*^21^, utilising an exhaustive search maximising the Pearson r correlation between a candidate rate map and the best fitting boundary vector cell model rate map. Specifically, we generated idealised boundary vector cell model rate maps according to the canonical Hartley *et al.*^18^ model, defining contributions to the boundary response, g, as the product of two Gaussians: one tuned to a preferred distance to the boundary, d, while the other was tuned to a preferred allocentric direction to the boundary, ω.


(4) $$g_{d,\omega }(r,\theta )\propto \frac{\text{exp}(\frac{-{\left(r-d\right)}^{2}}{2\sigma_{rad}^{2}(d)})}{\sqrt{2\pi \sigma_{rad}^{2}(d)}}\times \frac{\text{exp}(\frac{-{\left(\theta -\omega \right)}^{2}}{2\sigma_{ang}^{2}})}{\sqrt{2\pi \sigma_{ang}^{2}}}$$

Thus, for a boundary at distance, r, and allocentric direction, θ, subtending at an angle, δθ, the firing rate, f, of the boundary vector cell is given by:

(5) δf=g(r,θ)δθ

Note, $\sigma_{ang}$ is a constant while $\sigma_{rad}$ varies linearly with preferred distance tuning d:

(6) $$\sigma_{rad}(d)=\frac{d}{1+\beta }\sigma_{0}$$

for constant β and $\sigma_{0}$.

Following Muessig *et al.*^21^, we calculated idealised boundary vector cell firing for each position in a 25x25 unit grid, with constants $\sigma_{ang}=0.2$ radians and β=183 units fixed as used in Hartley *et al.*^18^. As with Muessig *et al.*^21^, we generated a total of N=3120 model cells, varying $\sigma_{0}=6.2,12.2,20.2$ or 30.2 units, with preferred distance tunings, d, ranging from d=1 to d=13 units in 1 unit increments, and preferred angular tunings, ω, varying from $\omega =0^{{}^{\circ}}$ to $\omega =354^{{}^{\circ}}$ in 6° increments.

In order to fit the boundary vector cell model to candidate rate maps (derived from either neural data or our successor representation model), we first resized each rate map, where necessary, in order to fit the 25x25 grid of the modelled boundary vector cells. We then performed resizing by MATLAB R2022b’s ‘imresize’ function with the method set to ‘bilinear’. Next, we performed a Pearson correlation between the candidate rate maps and each of the N=3120 boundary vector cell model ratemaps, identifying the d, ω, $\sigma_{0}$ parameters of the best fitting boundary vector cell. We classified a candidate rate map as a boundary vector cell if the maximum correlation across all model rate map fits exceeded r>0.7. To further analyse the four-fold symmetrical clustering of directional tuning preferences in the square environment, we collapsed direction tunings, ω, across all 4 walls, centred on the direction orthogonal to the wall, and quadrupled to span the full 360° before applying circular statistics^64^.

### Data preparation for behavioural skew analysis

In Figure. 3, we analysed the correlation between rodent’s behaviour and the development of asymmetry in subiculum. We performed the following analysis on CA1 neurons from Dataset 1 and subiculum neurons from Datasets 1 and 2, as these were the only two datasets that recorded neurons over consecutive and identical trials. Each ratemap was accompanied by an x/y vector of the rodent’s position over the corresponding trial.

Firstly, we z-scored each ratemap, ignoring NaNs, and set any negative values to 0. We then used scipy’s ‘ndimage’ package^65^ to identify the number of discrete objects in each ratemap. We discarded any ratemaps where we identified more than 5 objects.

To determine the relationship between the behavioural bias of the rodent and the skew of subiculum or CA1 fields in one-dimension, we limited our analysis to those cells whose primary firing field was <=5 bins of a boundary, where the rodent’s behaviour is more constrained (Fig.2d,e). This corresponds to <7.5cm for data from dataset 1 (N=42) and <8.3cm from dataset 2 (N=13). Dataset 3 did not include consecutive trials without other manipulations. If the number of objects identified was one, we took this object to be the main firing field. If the number was greater than one, we took the field with the highest maximum firing rate of the first three identified as the primary field. We then masked all of the ratemap except for this primary firing field in preparation for the next step.

Next, we chose the axis to collapse the newly masked ratemaps over (horizontal or vertical) as that which gave the lowest maximum when each ratemap was summed over it. Because many fields lay close to a corner and were therefore close to two walls, we only included those cells whose masked ratemaps collapsed over the same axis (horizontal or vertical) in both trials. We calculated the centre of mass of each masked ratemap, now averaged onto one axis, and used the change in this between two trials to quantify the overall change in field skew.

For the behavioural skew analysis, we first isolated the subsections of the animal’s trajectory that ran through each cell’s primary firing field (defined above). We then calculated the resulting allocentric direction between each consecutive position, if that distance was >0.0001 along either the x or y dimension (i.e. the agent moved >0.025cm on either axis). We collected these along the whole trajectory and binned them into a single histogram for each trial, between the allocentric angles of [-π∕4, π∕4, π3∕4, π5∕4] as up, right, left and down. We calculated the overall behavioural skew of the animal’s movement through this firing field by subtracting the size of the histogram bar for the ‘backwards’ direction (left or down for fields running along horizontal and vertical walls respectively) from the ‘forwards’ direction (rightwards or upwards for fields running along the horizontal and vertical walls respectively).

Figure. 3e,f presents the correlation of the change in the skew of each cell’s main field against the rodent’s change in behavioural bias through that field between the two trials. In order to exclude any cells that are unstable between trials, we only included filtered ratemaps (displaying only the primary field) with at least a 0.75 correlation between trials 1 and 2, however using other thresholds did not significantly alter the results.

### Corner score analysis

For our corner cell analysis, we repeated the procedure of Sun *et al.*^9^ for square, hexagon, triangle and circular-shaped environments. Since it is unclear how Equation 3 can be extrapolated to non-rectangular environments, we generated populations of 100 symmetric Gaussian place cell basis features, with standard deviation 6cm, and calculated the successor features based on the longest distance rodent trajectory for each geometry, as outlined in Section 1. Following Sun and colleagues^9^, we thresholded each successor representation and place cell ratemap at 30-40% of their maximum value to best isolate their main firing fields. Next, we set all values of these ratemaps that were negative to be 0, and performed object labelling using scipy’s ‘ndimage’^65^. For each identified object in each ratemap, we calculated a corner score based on the distances between the object’s centroid, the environment’s centre, d1, and the nearest corner, d2, as per Sun *et al.*^9^:

(7) $$cornerscore_{field}=\frac{d_{1}\;-\;d_{2}}{d_{1}\;+\;d_{2}}$$

For ratemaps where the number of identified objects was less than the number of corners, we calculated the overall corner score of a ratemap with k corners and n fields as per Sun *et al.*^9^:

(8) $$cornerscore_{cell}=\frac{\sum_{n}cornerscore_{field}}{k},(n\leq k)$$

For ratemaps where the number of identified objects was greater than the number of corners, k, we calculated the corner score using the top n scores as:

(9) $$cornerscore_{cell}=\frac{\sum^{k}cornerscore_{field}\;-\;\sum_{k}^{n}|cornerscore_{field}\;-\;1|}{k},(n>k)$$

In order to compare the distribution of corner scores between subicular cells and successor features, we generated 2000 shuffled successor features and took the 95th percentile of their corner scores as a benchmark. We calculated these shuffled successor features for each geometry by randomly shuffling the columns of our transition matrix, M, after it had been trained using the rodent’s trajectory, and re-generating 2D successor features based on this shuffled matrix. As per Sun *et al.*^9^, we did not include the punishment term for extra firing fields when we calculated the corner scores for shuffled successor features. Figure. 4f compares the percentage of successor features and the percentage of place cells that were classified as corner cells by this shuffled threshold, to the percentage of corner cells that are reported for each geometry in Sun *et al.*^9^, using their own spike-train shuffle threshold.

## Supplementary Material

Supplement 1

## Data Availability

The neural recordings analysed in this study were obtained from previously published datasets (Muessig et al. (2015), Muessig et al. (2019), Poulter et al. (2021), Muessig et al. (2024), Sun et al. (2024)). Ownership and responsibility for the dissemination of this data remains with the original authors.

## Code availability

The code required to replicate the analysis in this study is publicly available at: https://github.com/Lauren2909/Unifying-Manuscript.
