# Supplementary material for: Unifying Subicular Function: A Predictive Map Approach
Source: bioRxiv. 2024 Nov 7:2024.11.06.622306. Preprint. [Version 1] doi: 10.1101/2024.11.06.622306 (PMC11580870; doi:10.1101/2024.11.06.622306)
Supplement: Supplementary file 1 [file media-1.pdf]

Supplementary information

Additional RSA results

Figure 6 provides further RSA comparisons with other candidate models. Importantly, subicular cells are also well fit by successor features that are built on symmetrical and uniform Gaussian basis features, which do not deform in size and shape around boundaries. Further, all subicular recordings are significantly less well modelled by both traditional boundary vector cells[18], and uniform, symmetric Gaussian basis features, compared to successor features built on biological basis features and real rodent trajectories.

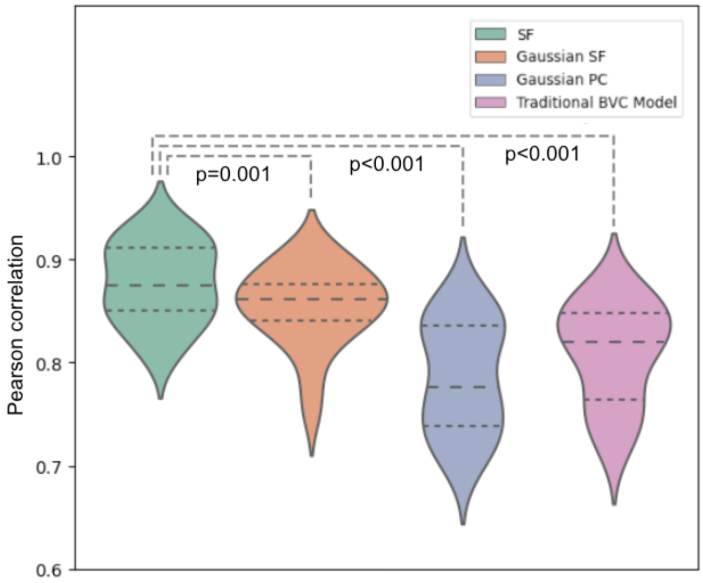

Fig. 6 Additional RSA correlations to subiculum recordings. All subicular recordings are better fit by successor features built on biologically-scaled basis features and real rodent trajectories, than successor features built on uniform and symmetric Gaussian place cell bases and real rodent trajectories. However, all subicular recordings are distinctly better fit by both populations of successor features than either Gaussian basis features, or traditional 'boundary vector cells'[18].
